# Prognostic value of *SLC4A4* and its correlation with the microsatellite instability in colorectal cancer

**DOI:** 10.3389/fonc.2023.1179120

**Published:** 2023-04-19

**Authors:** Shaorui Rui, Dong Wang, Yong Huang, Jingyun Xu, Hailang Zhou, Hesong Zhang

**Affiliations:** ^1^ Department of General Surgery, The Second Affiliated Hospital of Wannan Medical College, Wuhu, China; ^2^ Department of Hepatobiliary Surgery, The First Affiliated Hospital of Wannan Medical College, Wuhu, China; ^3^ School of Basic Medicine, Wannan Medical College, Wuhu, China; ^4^ Department of Gastroenterology, Lianshui People’s Hospital Affiliated to Kangda College of Nanjing Medical University, Huai’an, China; ^5^ The Institute of Life Sciences, Jiangsu College of Nursing, Huai’an, China; ^6^ Department of Hepatobiliary Surgery, The Second People’s Hospital of Wuhu, Wuhu, China

**Keywords:** microsatellite instability, single-cell sequencing, colorectal cancer, biomarker, Slc4a4

## Abstract

**Objective:**

To explore new biomarkers related to microsatellite instability in order to better predict prognosis and guide medication.

**Methods:**

The “limma” R package was used to identify differentially expressed genes in GSE24514, and then weighted correlation network analysis was used to select key genes. Different cell types in the tumor microenvironment were identified and analyzed by single-cell sequencing, with a Lasso regression model used to screen prognostic variables. Furthermore, the correlation between microsatellite instability and potential prognostic variables was explored, as well as the expression characteristics and clinical characteristics of the prognostic variables in the TCGA, UALCAN, and HPA databases. PCR assay was used to investigate the expression of *SLC4A4* in colorectal cancer cell lines. Finally, we further verified the expression of *SLC4A4* by immunohistochemistry.

**Results:**

First, 844 differentially expressed genes in GSE24514 were identified. Subsequently, weighted co-expression network analysis (WGCNA) of GSE24514 obtained all the genes significantly associated with microsatellite instability (MSI), a total of 1452. Analysis of GSE166555 single cell sequencing data set yielded 1564 differentially expressed genes. The gene sets obtained from the above three analysis processes were intersected, and 174 genes were finally obtained. The Lasso regression model revealed two potential prognostic genes, TIMP1 and *SLC4A4*, of which, there was a stronger correlation between microsatellite instability and *SLC4A4*. The mRNA and protein expression of *SLC4A4* was significantly decreased in tumors, and patients with low *SLC4A4* expression had a poor prognosis. In addition, *SLC4A4* was specifically expressed in epithelial cells. In the microenvironment of colorectal cancer, malignant cells have a strong interaction with different stromal cells. PCR showed that *SLC4A4* was significantly down-regulated in colorectal cancer cell lines Caco-2, HCT116 and HT29 compared with normal control NCM460 cell lines. Immunohistochemistry also showed low expression of *SLC4A4* in colorectal cancer.

**Conclusion:**

*SLC4A4*, as a tumor suppressor gene, is significantly downregulated and positively correlated with microsatellite instability, thus it may be combined with microsatellite instability to guide colorectal cancer treatment.

## Introduction

Colorectal cancer (CRC) is one of the most common gastrointestinal malignancies in the world, which is characterized by its high incidence and recurrence rate (1). The liver is the most common site of metastasis, and the 1-,3-and 5-year survival rates of patients with liver metastasis are far from satisfactory (2). According to statistics, there are more than 1.8 million new cases in the world every year (3). At present, due to the great progress in the pathophysiology of colorectal cancer, the treatment options have also increased, including endoscopic and surgical resection, radiotherapy, immunotherapy, targeted therapy, and local ablation (4–7). However, CRC is still the third most frequently diagnosed cancer and the second leading cause of cancer death worldwide due to imperfect screening programs, treatment strategies, and increased incidence (8). Therefore, identifying predictive biomarkers and revealing supporting mechanisms are urgently needed when predicting and treating CRC. The large-scale sequencing cancer genome project has identified biomarkers with potential clinical and therapeutic value, including microsatellite instability (MSI). In normal cells, the mismatch repair (MMR) system verifies and maintains the repeated count of microsatellites during cell division, which is one of the cellular DNA repair mechanisms. The damage to the MMR system causes cells to be unable to adjust the length of their microsatellite during cell division, known as MSI.

After several cell division cycles, the damaged cells will develop cells with different lengths of microsatellite sequences. MSI is often observed in colorectal cancer, endometrial cancer, and gastric adenocarcinoma and has been used in treating colorectal cancer with an improved prognosis of patients with MSI-H (MSI-high) colorectal cancer compared to patients with MSI-L (MSI-low) tumor. In addition, MSI-H colorectal tumors have been proven to be more susceptible to immune enhancement therapy. In 2019, FDA approved pembrolizumab for the treatment of patients with advanced MSI tumors (9), indicating that a programmed death 1 receptor (PD-1) blockade has become a highly relevant treatment choice for this patient group, regardless of tumor site or histology (10).

The solute carrier (SLC) family is a group of membrane transport proteins that play critical roles in the transportation of various metabolites, nutrients, and drugs across cell membranes. Dysregulation of SLC proteins has been implicated in the development and progression of various cancers, including breast, lung, prostate, and colorectal cancer (11). Studies have shown that alterations in SLC expression and activity can affect tumor cell growth, survival, and metastasis.

For instance, SLC transporters have been found to play a role in the uptake of nutrients such as glucose and amino acids, which are essential for cancer cell metabolism and growth. The upregulation of SLC transporters, such as SLC7A5 and SLC1A5, has been observed in various cancer types and is associated with poor prognosis. In contrast, the downregulation of SLC transporters, such as SLC26A4 and SLC5A8, has been shown to inhibit cancer cell proliferation and migration (12, 13).

The bicarbonate transporter consists of two families, SLC4 and SLC26, which can be further subdivided into acid loaders or acid extruders depending on the orientation of the transporter (14). The acidic extruder absorbs bicarbonate and thus prevents TME acidification by acting on the acidic extruder. The reduced expression of *SLC4A4* can promote cancer cell proliferation and migration traits *in vitro* or under the condition of immunodeficiency, which is mainly dependent on the tumor cell type. In renal clear cell carcinoma, miR-223-3p promotes cell proliferation and metastasis by downregulating *SLC4A4* (15). This study investigated novel biomarkers that are significantly associated with MSI and have significant prognostic implications.

## Materials and methods

### Data download and processing

The TCGA-COAD cohort data, including gene expression data from 471 tumor tissues, survival data from 454 patients, clinical phenotype data from 478 patients, and tumor mutation data from 399 patients, were obtained from the UCSC Xena website (http://xena.ucsc.edu/). The high-throughput sequencing data GSE24514 was downloaded from the GEO database. There were 49 samples in total, and 44 samples remained after quality control. Single cell sequencing data GSE166555 was downloaded from the GEO database and included 12 tumor tissues and their paracancer controls. All the data was log2 transformed.

### Differential gene expression by microarray data mining

The expression data of GSE24514 were downloaded from the GEO database (16) and then, principal component analysis (PCA) and UMAP (Uniform Manifold Approximation and Projection for Dimension Reduction) were used to visualize each sample in groups and remove outliers. The R software package limma (version 3.40.6) was then used to identify the differentially expressed genes. Briefly, the data was log2 transformed and subjected to multiple linear regression using the lmFit function. We set up | log2FC | > 1 and *P* - value < 0.05. The differentially expressed genes were visualized in volcano maps and thermal maps.

### Identification of candidate biomarker gene by WGCNA

WGCNA (Weighted Correlation Network Analysis) is a systematic biological method used to describe gene association patterns between different samples to identify gene sets with highly synergistic changes and candidate biomarker genes or therapeutic targets based on the interconnectivity of gene sets and the association between gene sets and phenotypes. When compared to focusing only on differentially expressed genes, WGCNA identified the gene set of interest using the information regarding thousands or nearly ten thousand of the most varied genes or all genes and performed significant association analysis with phenotypes.

### Single-cell sequencing to explore heterogeneity in the tumor microenvironment

The sequencing data from TISCH2 and GSE166555 from the GEO database were used to characterize the tumor microenvironment at the single-cell resolution (17). Several different cell types were identified after dimensionality reduction clustering and annotation of cell markers. Understanding the cell-cell interaction (CCI) is essential to study how these cells and signals coordinate function, therefore CellChat was integrated to infer the cell-cell communications between each cluster (18, 19).

### Screening potential biomarkers using the Lasso regression model

The RNAseq data was downloaded from the TCGA database (https://portal.gdc.cancer.gov) for the STAR process of the TCGA project and the data was extracted in TPM format and the clinical data. Additional prognostic data was obtained from the literature (20). The data processing method is log2(value+1) and the cleaned data was analyzed using the “glmnet” R package to obtain the variable lambda value, maximum likelihood number, or C index (21).

### The correlation between genes and MSI

MSI occurs due to a functional defect in the DNA mismatch repair of tumor tissue and is an important tumor marker. The unified and standardized pan-cancer data set was downloaded from the UCSC (https://xenabrowser.net/) database, and the expression data of ENSG00000080493 (*SLC4A4*) was extracted. The MSI score for each tumor was obtained from a previous study (22) and integrated with the gene expression data to calculate the Pearson correlation for each tumor.

### Identification of expression characteristics and clinical features of *SLC4A4*

First, the difference in genes between the two groups was identified based on the TCGA data using the Wilcoxon rank sum test. They were then visualized using the “ggplot2” package. *SLC4A4* transcription and protein levels, as well as survival curves, were evaluated in the UALCAN database (23). The time-dependent AUC is a polygonal line that shows the area (AUC) under the corresponding curve of different variables at different times and is < 0.5 when the value of the variable (protective factor) is opposite to the trend of the event. The AUC of ROC is often used for the evaluation of diagnostic tests and generally, an AUC within the range of 0.5 and 1, and closer to 1 indicates that the variable has a better diagnostic effect on the predicted outcome. The data was analyzed using the timeROC package and the pROC package, and the results were visualized using ggplot2. The *SLC4A4* transcriptome data was combined with clinical data, and grouping comparisons and survival curves were drawn to describe differences between the clinical groupings. The *SLC4A4* proteomic profile was validated using immunohistochemical images from the HPA database (24).

### Cell culture

Suzhou Medical University (Suzhou, China) provided the following human colon cancer cell lines: HT29, Caco-2, and HCT116, as well as the normal colon cell line NCM460. There were cultured in DMEM (HyClone, USA) supplemented with 10% FBS (Gibco, USA) and 100 µg/ml streptomycin/penicillin (Hyclone) in a 5% CO2 humidified environment at 37°C. The cells were passaged every 2–3 days using 0.25% trypsin (Hyclone).

### RT-PCR

The expression of *SLC4A4* in different colon cancer cell lines was quantified by qRT-PCR, with at least three biological replicates per sample. The total RNA concentration of each sample was adjusted to be the same before reverse transcription using the ChamQ Universal SYBR qPCR Master Mix and HiScript II Q RT SuperMix for qPCR (Nanjing Novozan Biotechnology Co. ltd). The relative mRNA expression was calculated using the 2ΔΔCT method and normalized to the internal reference β-actin. The primer sequences were as follows:

*SLC4A4*: Forward: 5′-TTGCCAACTATGTCTTCTACTGA-3′
Reverse: 5′-ATTACAGTTGTTCCCGACGAG-3′
β-actin: Forward: 5′-GTGGCCGAGGACTTTGATTG-3′
Reverse: 5′-CCTGTAACAACGCATCTCATATT-3′

### Immunohistochemistry

Tissue chips are used for Immunohistochemistry (IHC) which was performed as per standard protocols. In summary, the tissue samples were fixed in 4% paraformaldehyde, embedded in paraffin, and sectioned. Following deparaffinization, rehydration, antigen retrieval and blocking, incubation of the slides with primary antibodies was conducted at 4°C. The sections then underwent incubation with an HRP-conjugated anti-rabbit secondary antibody (Servicebio, China). The chromogen used was diaminobenzidine (DAB). To capture the images under white light, a fluorescence microscope from Olympus (Japan) was used.

### Statistical analysis

The analysis was conducted using R software (version 4.1.0). The Wilcoxon test was employed to compare the two groups, while the Kruskal-Wallis test was used for comparing multiple groups. A p-value of < 0.05 was considered statistically significant. The Spearman correlation test was utilized to compare the correlations between two continuous variables.

## Results

**Figure 1:**
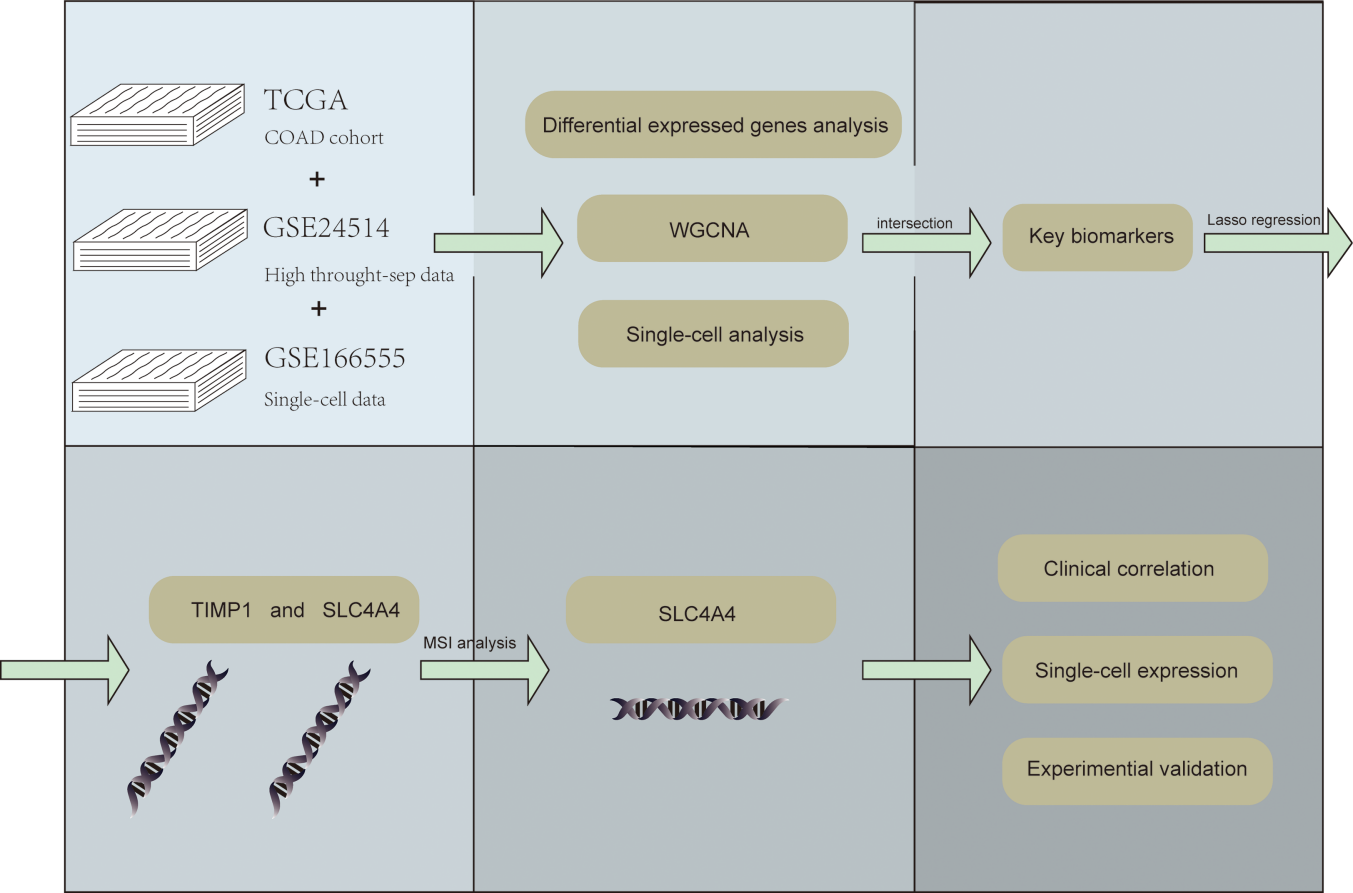
The flow chart.

**Figure 1** shows the workflow of our study.

### Data quality control and identification of differentially expressed genes

**Figure 2:**
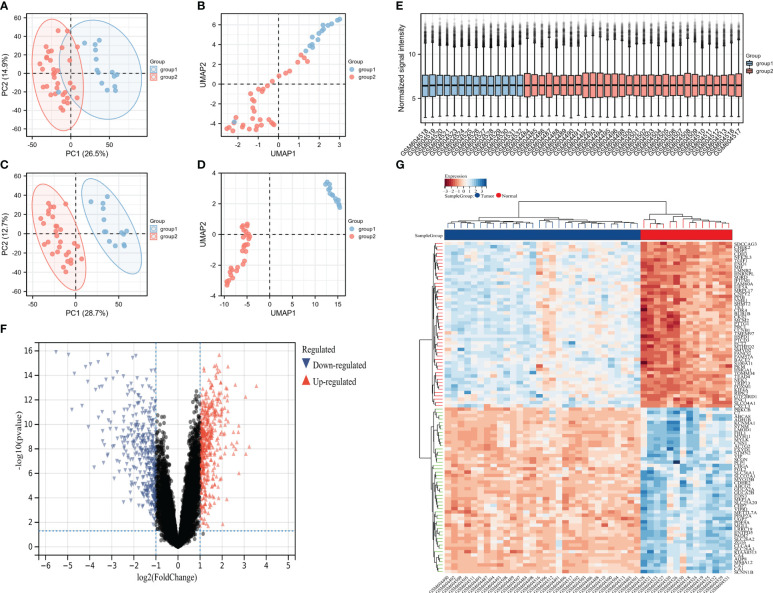
The sample quality was acceptable and many differential genes were identified. **(A)** PCA before quality control, **(B)** UMAP before quality control, **(C)** PCA after quality control, **(D)** UMAP after quality control, **(E)** sample normalization box diagram, **(F)** volcanic map, and **(G)** heat map.

First, the spatial features in the PCA map and the UMAP map were used to represent the biological characteristics of each sample and significant inter-group differences were observed after the removal of the lower-quality samples (**Figures 2A–D**). The sample normalization box plot showed good correction for all samples and no significant batch effects or other rejected samples (**Figure 2E**). Subsequently, volcanic and thermal maps were used to display the 844 differential genes meeting the threshold, including 648 genes with significantly high expression and 216 genes with significantly low expression (**Figures 2F, G**).

### Identification of MSI-associated candidate biomarker genes in colorectal cancer by WGCNA

**Figure 3:**
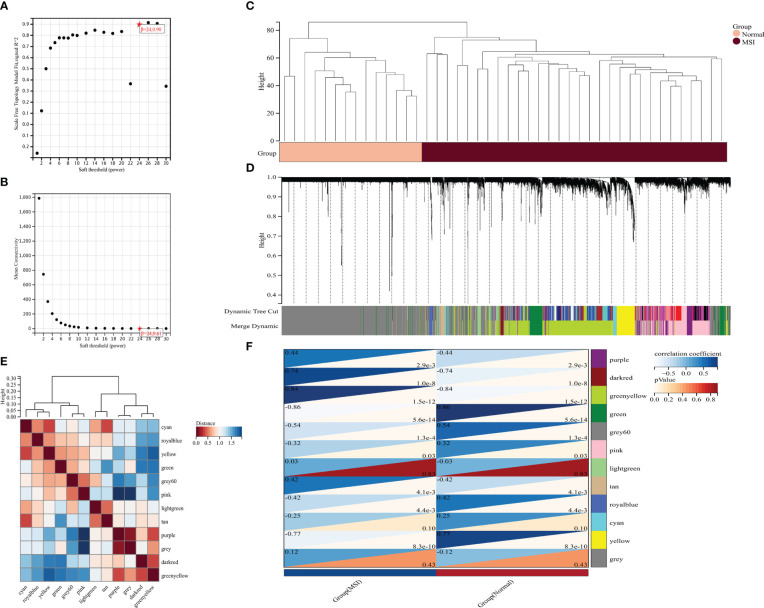
Mining potential biomarker genes associated with MSI based on weighted gene networks. **(A)** scale independence, **(B)** average connectivity, **(C)** The sample clustering indicated no outlier samples, **(D)** gene clustering, **(E)** module feature vector clustering, and **(F)** heat map of module and phenotype correlation.

A weighted gene network was constructed to identify candidate biomarkers associated with MSI in colorectal cancer. Scale independence and mean connectivity indicated that the soft threshold used to construct the weighted gene network was 24 (**Figures 3A, B**). Sample clustering revealed significant differences between the two groups, and there were no outliers (**Figures 3C, D**). The hierarchical clustering diagram identified gene modules with high correlation, with the clustering heat map drawn according to the different vector features of each module, showing the distance between the different modules (**Figure 3E**). Finally, association analysis was performed on each module and phenotype to identify modules with a high correlation with the phenotype of interest and then the key genes were extracted from the statistically significant modules (**Figure 3F**).

### Single cell sequencing revealed tumor heterogeneity and characteristics of different cell groups in colorectal cancer

**Figure 4:**
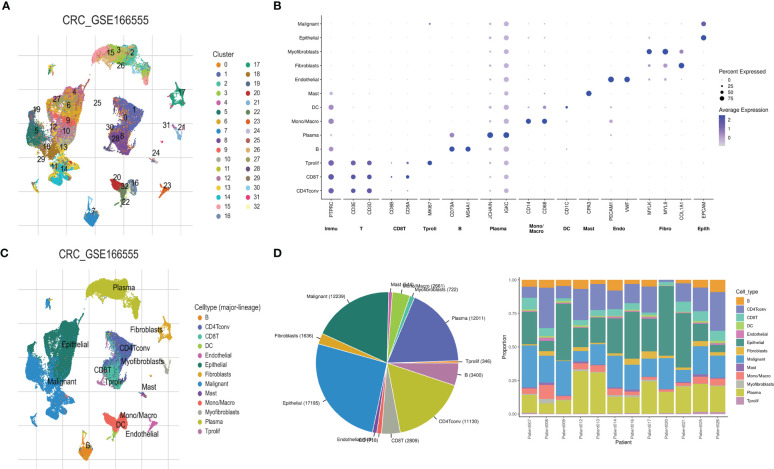
Cell heterogeneity in the microenvironment of colorectal cancer revealed by single-cell sequencing. **(A)** The UMAP map revealed 33 different cell clusters, **(B)** cell marker dot pattern, **(C)** 13 cell types were obtained after cell annotation, and **(D)** fan and stacked bar graphs show the proportion of the different cell types.

Single-cell sequencing revealed 33 different cell clusters (**Figure 4A**) in the GSE166555 dataset through dimensionality reduction and clustering. The punctiform figures show the cell marker for cell annotation and their expression levels (**Figure 4B**). Annotating these clusters through cell markers yielded 13 cell types, Including B cells, CD4Tconv cells, CD8T cells, DC cells, endothelial cells, epithelial cells, fibroblasts, malignant cells, mast cells, Mono/Macro cells, myofibroblasts, plasma tumors, and Tprolif cells (**Figure 4C**). The pie chart showed the proportion of the different types, and the stacked histogram showed the proportion of the different types in each sample (**Figure 4D**).

### Potential biomarkers identified by Lasso regression

**Figure 5:**
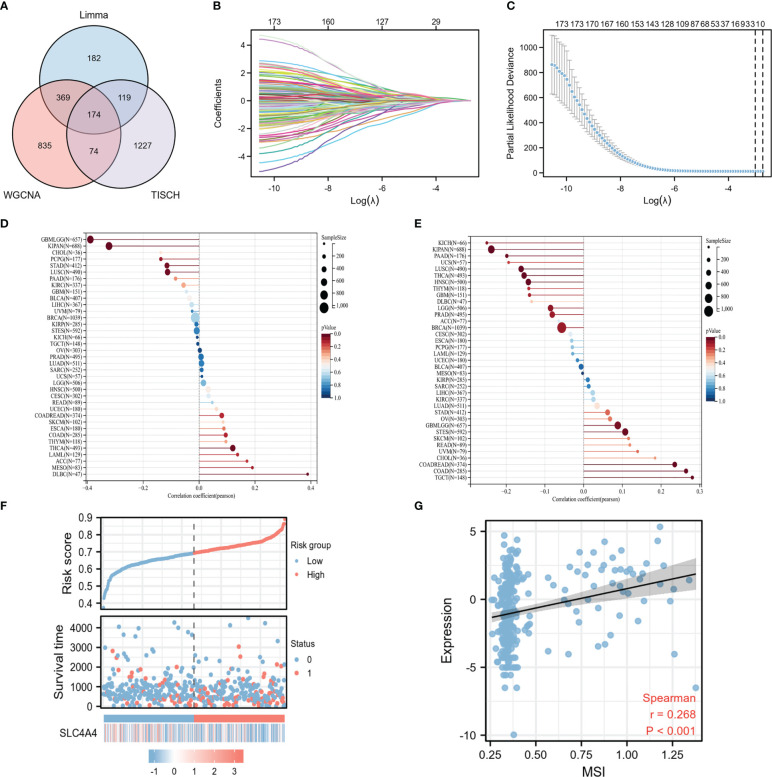
The correlation between the potential biomarker *SLC4A4* and MSI is significant. **(A)** The Venn diagram indicates 174 intersecting genes, **(B)** the trace diagram of the different variables in the Lasso regression, **(C)** two undetermined variables were obtained by Lasso regression, **(D)** lollipop plot of the correlation between TIMP1 expression in different tumors and MSI, **(E)** the lollipop plot of correlation between *SLC4A4* expression in different tumors and MSI, **(F)** risk factor diagram, and **(G)** correlation scatter plot.

Intersection analysis was performed of the results of the limma difference analysis, WGCNA, and single-cell sequencing difference analysis to construct a Venn diagram to obtain 174 intersection genes (**Figure 5A**). Subsequently, these 174 crossed genes were further screened using the Lasso regression model, and two potential biomarkers were identified: TIMP1 and *SLC4A4* (**Figures 5B, C**). To explore which gene had a stronger association with MSI, a lollipop graph of the association between gene expression and MSI was drawn showing that the association between *SLC4A4* and MSI was significantly higher in colorectal cancer than in TIMP1 (**Figures 5D, E**). According to the risk factor map, when *SLC4A4* expression is decreased, the risk score is significantly increased, and the prognosis is poor (**Figure 5F**). *SLC4A4* expression and the MSI for each sample were visualized in the correlation scatter plot and the correlation coefficient was 0.268 (**Figure 5G**).

### Expression and clinical characteristics of the MSI-related gene *SLC4A4*

**Figure 6:**
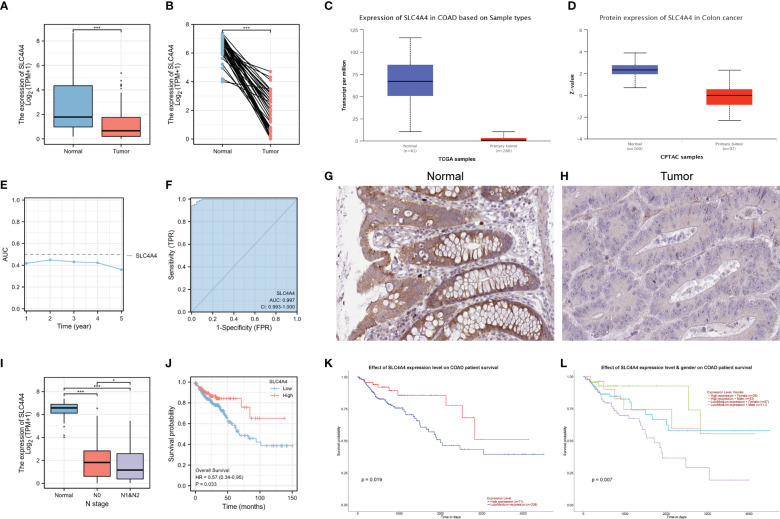
*SLC4A4* is significantly downregulated in tumors and contributes to poor prognosis. **(A)** Non-paired samples, **(B)** paired samples, **(C)** *SLC4A4* transcriptional levels in UALCAN-TCGA, **(D)** *SLC4A4* protein levels in UALCAN-CPTAC, **(E)** time-dependent AUC, **(F)** ROC tested the stability of *SLC4A4* as a biomarker, **(G)** *SLC4A4* protein expression in normal tissue, **(H)** *SLC4A4* protein expression in tumor tissues, **(I)** the difference in *SLC4A4* expression in different N stages, **(J)** low *SLC4A4* expression is associated with poor prognosis, **(K)** the low *SLC4A4* expression in the UALCAN database contributed to poor prognosis, and **(L)** grouping in combination with gender revealed a significantly poor prognosis for men with low *SLC4A4* expression. *P<0.05; ***P<0.001.

*SLC4A4* mRNA and protein expression were significantly downregulated in tumors in both the TCGA and UALCAN databases (**Figures 6A–D**). The time-dependent AUC suggested that *SLC4A4* might be a protective factor and ROC indicated that *SLC4A4* is highly sensitive and specific (**Figures 6E, F**). The immunohistochemical results highlighted the decreased *SLC4A4* expression in the tumor (**Figures 6G, H**). Also, there was lower *SLC4A4* expression in patients with lymphoid metastasis (**Figure 6I**) and low *SLC4A4* expression was associated with poor prognosis (**Figures 6J–L**).

### High *SLC4A4* expression in epithelial cells

**Figure 7:**
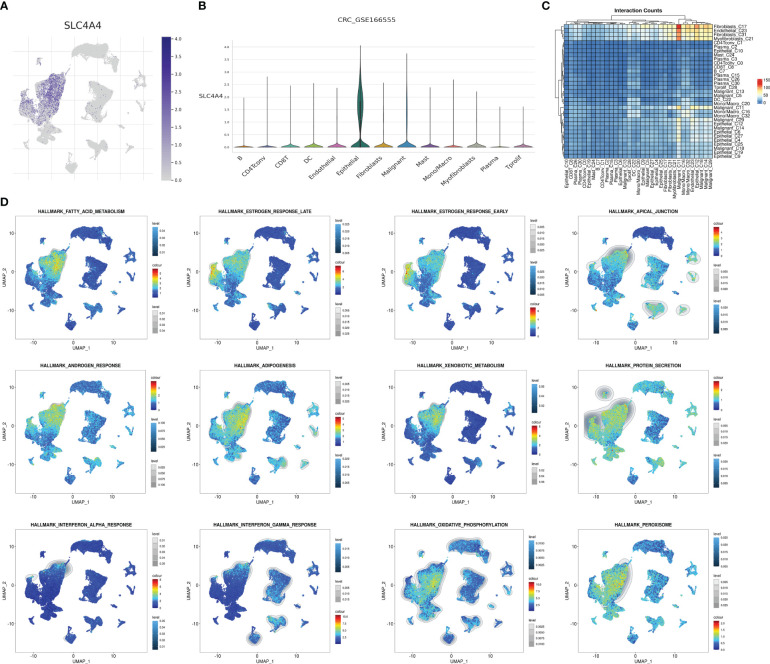
*SLC4A4* is specifically and highly expressed in epithelial cells. **(A)** The UMAP plot shows *SLC4A4* expression in each cell type, **(B)** the violin diagram shows *SLC4A4* expression in each cell type, **(C)** cell communication heat map, and **(D)** enrichment intensity and the fraction of different hallmark signaling pathways in each cell.

Using previous single-cell sequencing data to visualize *SLC4A4* expression in different cells revealed that *SLC4A4* expression was significant in epithelial cells and barely expressed in other cells, including malignant tumor cells (**Figures 7A, B**). Malignant cells strongly interacted with myofibroblasts, fibroblasts, and endothelial cells (**Figure 7C**). Also, some pathways were significantly enriched in epithelial cells or malignant tumor cells including fatty acid metabolism, estrogen response late, estrogen response early, apical junction, androgen response, adipogenesis, xenobiotic metabolism, protein secretion, interferon-alpha response, interferon-gamma response, oxidative phosphorylation, and peroxisome (**Figure 7D**).

### PCR and immunohistochemistry to validate the expression of *SLC4A4*

**Figure 8:**
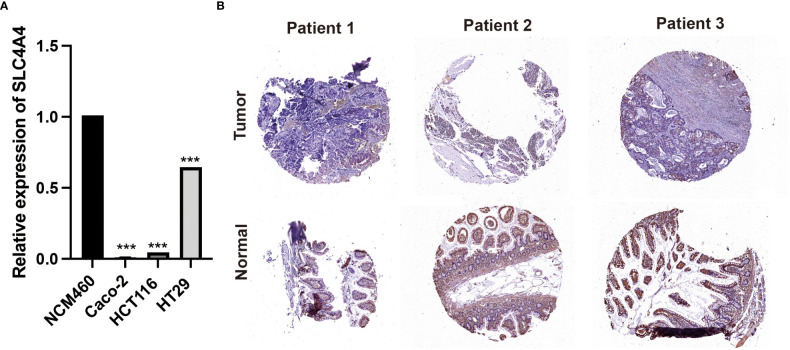
Experimental verification of *SLC4A4*. **(A)** PCR experiments in cell lines. *SLC4A4* was significantly down-regulated in colorectal cancer cell lines Caco-2, HCT116 and HT29 (***P<0.001). **(B)** Immunohistochemical examination of 3 clinical samples. The expression of *SLC4A4* is low in tumor tissue.

In order to further verify the expression of *SLC4A4*, we first performed PCR experiments on cell lines. The results showed that *SLC4A4* was significantly down-regulated in colorectal cancer cell lines Caco-2, HCT116 and HT29 compared with normal control NCM460 cell lines (**Figure 8A**, ***P<0.001). Subsequently, clinical samples of colorectal cancer from 3 patients were collected for immunohistochemical experiments. It can be seen that *SLC4A4* expression was downregulated in the tumor tissues of three patients compared with the adjacent normal tissues (**Figure 8B**).

## Discussion

Recently, the relationship between microsatellite instability and the occurrence and development of tumors has become a topic of interest in the study of tumor markers, characteristics, and prognosis. Although there have been many in-depth studies on potential biomarkers of tumors, few biomarkers can be used in combination with MSI to evaluate patient prognosis (25–28). MSI is a feature of many disorders, most of which are neoplastic, with Lynch syndrome being the most well-known non-neoplastic disorder. Protein dysfunction in the MMR family is involved in the occurrence of Lynch syndrome, with most families diagnosed with Lynch syndrome having MLH1 and MSH2 mutations, some families having MSH6 mutations, and a few families having PMS2 mutations (29). The occurrence of Lynch syndrome and partial sporadic colorectal cancer is not related to oncogene activation and tumor suppressor gene inactivation, rather it is caused by MSI due to mutations in mismatch repair genes (30). Currently, MSI/dMMR detection is an important diagnostic indicator for screening patients with Lynch syndrome. In summary, MSI has important clinical implications for Lynch syndrome screening, predicting the prognosis of colorectal cancer, and guiding drug use.

In recent years, significant progress has been made in the field of bioinformatics for CRC research (31, 32). Studies have employed various bioinformatics tools, including transcriptomics, genomics, proteomics, and metabolomics, to investigate the molecular mechanisms underlying CRC development and progression (33). For instance, transcriptomic profiling of CRC tissues has identified gene expression signatures associated with different stages of CRC, providing potential biomarkers for early diagnosis and personalized treatment (34). Additionally, genomic studies have revealed somatic mutations and genetic alterations associated with CRC, including the well-known APC, TP53, and KRAS mutations (35). Other studies have utilized proteomic and metabolomic approaches to identify protein and metabolite biomarkers for CRC diagnosis and prognosis. The integration of multi-omics data has also been increasingly used to improve the accuracy of CRC diagnosis and prognosis (36).

Here, for the first time, we identified a significant positive correlation between *SLC4A4* and MSI in colorectal cancer. Potential genes were screened using second-generation sequencing data, weighted average co-expression network, and single-cell sequencing data to obtain *SLC4A4* using Lasso regression and MSI correlation calculation. It is well known that patients with MSI-H have a better prognosis than MSS despite a poorer clinical presentation.

We identified a significant positive correlation between MSI and *SLC4A4* expression, the significant downregulation of *SLC4A4* mRNA and protein expression in tumors, and the *SLC4A4* low-expression group had a poorer prognosis which corresponds to MSI. More importantly, *SLC4A4* expression in the N1–N2 group was much lower than that in N0, suggesting that *SLC4A4* might also mediate tumor cell metastasis to lymph nodes. Single-cell sequencing revealed that *SLC4A4* was specifically and highly expressed in epithelial cells. In UMAP, the spatial relative positions of different cell types represent the similarities between biological functions, such as T cells and their subsets, and the close relationship between malignant tumor cells and epithelial cells suggests that malignant tumor cells may evolve from epithelial cells, while *SLC4A4* is rarely expressed in malignant tumor cells. In our analysis, *SLC4A4* was localized on the plasma membrane and involved in the regulation of bicarbonate secretion and absorption as well as intracellular pH. It is hypothesized that the absence of *SLC4A4* may lead to an imbalance between intracellular pH and carcinogenesis.

## Conclusion

For the first time, we have identified *SLC4A4* as a potential prognostic biomarker significantly associated with MSI and related to the intracellular pH. The combination of *SLC4A4* and MSI can predict the prognosis and outcomes of colorectal cancer patients and guide clinical medication, providing innovative recommendations for personalized medicine.

## Data availability statement

The original contributions presented in the study are included in the article/supplementary material. Further inquiries can be directed to the corresponding authors.

## Ethics statement

The studies involving human participants were reviewed and approved by The Second Affiliated Hospital of Wannan Medical College. The patients/participants provided their written informed consent to participate in this study.

## Author contributions

All authors contributed to the study conception and design. Material preparation, data collection and analysis were performed by YH, JX and HaZ. The first draft of the manuscript was modified by SR, DW and HeZ. The revision draft of the manuscript was written by SR and HeZ. All authors commented on previous versions ofthe manuscript. All authors read and approved the final manuscript.
